# Role of SIRT3 in neurological diseases and rehabilitation training

**DOI:** 10.1007/s11011-022-01111-4

**Published:** 2022-11-14

**Authors:** Yanlin Li, Jing Li, Guangbin Wu, Hua Yang, Xiaosong Yang, Dongyu Wang, Yanhui He

**Affiliations:** 1 Department of Rehabilitation, Jinzhou Central Hospital, 51 Shanghai Road, Guta District, Jinzhou, 121000 Liaoning Province People’s Republic of China; 2 Department of Neurology, Jinzhou Central Hospital, 51 Shanghai Road, Guta District, Jinzhou, 121000 Liaoning Province People’s Republic of China; 3 Department of Radiology, Jinzhou Central Hospital, 51 Shanghai Road, Guta District, Jinzhou, 121000 Liaoning Province People’s Republic of China

**Keywords:** SIRT3, Deacetylase, Neurological diseases, Rehabilitation training

## Abstract

Sirtuin3 (SIRT3) is a deacetylase that plays an important role in normal physiological activities by regulating a variety of substrates. Considerable evidence has shown that the content and activity of SIRT3 are altered in neurological diseases. Furthermore, SIRT3 affects the occurrence and development of neurological diseases. In most cases, SIRT3 can inhibit clinical manifestations of neurological diseases by promoting autophagy, energy production, and stabilization of mitochondrial dynamics, and by inhibiting neuroinflammation, apoptosis, and oxidative stress (OS). However, SIRT3 may sometimes have the opposite effect. SIRT3 can promote the transfer of microglia. Microglia in some cases promote ischemic brain injury, and in some cases inhibit ischemic brain injury. Moreover, SIRT3 can promote the accumulation of ceramide, which can worsen the damage caused by cerebral ischemia–reperfusion (I/R). This review comprehensively summarizes the different roles and related mechanisms of SIRT3 in neurological diseases. Moreover, to provide more ideas for the prognosis of neurological diseases, we summarize several SIRT3-mediated rehabilitation training methods.

## Introduction

The Sirtuins family contains seven members, which are marked as SIRT1 to SIRT7 (Chang and Guarente 2014; Imai and Guarente 2014; Kane and Sinclair 2018). The members of this family participate in a variety of physiological activities and pathological reactions by causing different types of post-translational modifications of various substrate proteins (Kida and Goligorsky 2016; Morigi et al. 2018). The location of each type of sirtuin in the cell is not the same. SIRT2 is mainly located in the cytoplasm; SIRT1, SIRT6, and SIRT7 are mainly located in the nucleus; and SIRT3, SIRT4, and SIRT5 are mainly located in mitochondria (Chen et al. 2021a, b). As a deacetylase located in mitochondria, SIRT3 must have a relationship with mitochondrial function. According to the available literature, SIRT3 can directly regulate about 100 downstream proteins, and most of the substrate proteins are activated by SIRT3 deacetylation (Yang et al. 2016). The related physiological functions include regulation of material metabolism, maintenance of mitochondrial stability, apoptosis, and autophagy (Zhang et al. 2020a, b, c, d).

At present, neurological diseases are increasingly affecting the quality of life of the elderly, and mitochondrial function is closely related to neurological diseases (Carrì et al. 2018; Chan 2020; Todorova and Blokland 2017). The current research mainly concentrates on SIRT1 and SIRT2, and there are relatively few studies on SIRT3. Therefore, in this review, we summarize the different roles and related mechanisms of SIRT3 in neurological diseases and summarize several rehabilitation training methods related to SIRT3 that can improve the prognosis of neurological diseases.

## Deacetylation activity and function of SIRT3

### SIRT3 and substance metabolism

The main function of SIRT3 is to participate in the regulation of substance metabolism (Sidorova-Darmos et al. 2018) (Table 1). Normal material metabolism maintains the stability of the body, and its main function is to provide energy for life activities and generate metabolic wastes that are easy to eliminate through biotransformation.

**Table 1:** Known targets of SIRT3 and function

Function	Downstream protein	References
Substance Metabolism		
Glucose metabolism	CypD	(Wei et al. 2013a, b)
	LDHA	(Cui et al. 2015)
	MPC1	(Liang et al. 2015)
	PDH	(Jing et al. 2013)
	PDP1	(Fan et al. 2014)
	CS	(Cui et al. 2017)
	IDH2	(Smolková et al. 2020)
	SDH	(Li et al. 2016a, b)
Fatty acid metabolism	Aco	(Fernandes et al. 2015)
	ACSF3	(Sun et al. 2020)
	CACT	(Giangregorio et al. 2017)
	LCAD	(Hirschey et al. 2010)
	VLCAD	(Zhang et al. 2015)
	β-HAD	(Alrob et al. 2014)
	ECHS1	(Zhang et al. 2017)
	ACC1	(Xu et al. 2020)
OXPHOS	NDUFA9	(Ahn et al. 2008)
	SDHA	(Cimen et al. 2010)
	UQCRQ	(Sun et al. 2019)
	COX-1	(Tu et al. 2019)
	ATP synthase β	(Rahman et al. 2014)
	p53	(Lee et al. 2018)
	LRP130	(Liu et al. 2014)
	CerS1, CerS 2, CerS 6	(Novgorodov et al. 2016)
Amino acid metabolism	GDH	(Choi et al. 2016)
	OTC	(Hallows et al. 2011)
	CPS1	(Li et al. 2016a, b)
Non-nutrients metabolism	ALDH2	(Wei et al. 2013a, b)
	AceCS2	(Hallows et al. 2006)
Mitochondrial dynamics		
Mitochondrial fusion	Opa1	(Samant et al. 2014)
	LKB1	(Pillai et al. 2010)
Mitochondrial fusion	FOXO3a	(Tseng et al. 2013)
	LKB1	(Mao et al. 2022; Xue et al. 2019)
Mitochondrial biogenesis	LKB1	(Fu et al. 2012; Li et al. 2017)
	TFAM	(Liu et al. 2018a, b, c)
Mitophagy	FOXO3a	(Ma et al. 2018; Yu et al. 2017)
*OS*	NDUFA9	(Ahn et al. 2008)
	SDHA	(Cimen et al. 2010)
	UQCRQ	(Sun et al. 2019)
	COX-1	(Tu et al. 2019)
	ATP synthase β	(Rahman et al. 2014)
	p53	(Lee et al. 2018)
	CerS1, CerS2, CerS6	(Novgorodov et al. 2016)
	MnSOD	(Qiu et al. 2010)
	CAT	(Wang et al. 2014)
	PRDX3	(Wang et al. 2020a, b)
	FOXO3a	(Tseng et al. 2014; Zhang et al. 2018a, b)
	FOXO1	(Zhang et al. 2013)
	Gpx	(Yoon and Kim 2016)
	IDH2	(Someya et al. 2010)
	MTHFD2	(Wan et al. 2020)
	GOT2	(Yang et al. 2015a, b)
*Apoptosis*	GSK-3β	(Song et al. 2016)
	OGG1	(Cheng et al. 2013)
	Ku70	(Sundaresan et al. 2008)
	HSD17B10	(Liu et al. 2020a, b, c)
	CypD	(Liu et al. 2018a, b, c)
*Autophagy*	LKB1	(Zhang et al. 2018a, b)
	ATG5	(Liu et al. 2018a, b, c)

Glucose metabolism, lipid metabolism, and oxidative phosphorylation (OXPHOS) provide most of the energy required for life activities. During anaerobic oxidation of sugars, SIRT3 deacetylates cyclophilin D (CypD) (Wei et al. 2013a, b) and lactate dehydrogenase A (LDHA) (Cui et al. 2015), which in turn inhibit glycolysis and promote lactate production, respectively. During aerobic oxidation of sugars, SIRT3 can deacetylate and activate mitochondrial pyruvate carrier 1 (MPC1) (Liang et al. 2015) and pyruvate dehydrogenase (PDH) (Jing et al. 2013), which promote the entry of pyruvate into mitochondria and the production of acetyl-CoA, respectively. The activation of pyruvate dehydrogenase phosphatase 1 (PDP1) by SIRT3 can also activate PDH (Fan et al. 2014). SIRT3 promotes aerobic oxidation by activating multiple key enzymes in the tricarboxylic acid cycle (TCA) cycle, such as citrate synthase (CS) (Cui et al. 2017), isocitrate dehydrogenase 2 (IDH2) (Smolková et al. 2020), and succinate dehydrogenase (SDH) (Li et al. 2016a, b); in contrast, deacetylation of aconitase (Aco) by SIRT3 inhibits its activity and thus inhibits aerobic oxidation (Fernandes et al. 2015).

When the body is in a state of starvation, energy generated by fatty acid metabolism compensates for the lack of energy caused by insufficient sugar supply and maintains the body's homeostasis. First, acyl-CoA synthase family member 3 (ACSF3) is involved in the activation of fatty acids to produce acyl-CoA (Sloan et al. 2011), which is transported to mitochondria under the action of carnitine/acylcarnitine transporter (CACT). The deacetylation activation of ACSF3 and CACT by SIRT3 promotes this process (Giangregorio et al. 2017; Sun et al. 2020). Acyl-CoA in mitochondria undergoes β-oxidation to generate acetyl-CoA, which in turn participates in the TCA cycle. SIRT3 deacetylates and activates long-chain acyl-CoA dehydrogenase (LCAD) (Hirschey et al. 2010), very long-chain acyl-CoA dehydrogenase (VLCAD) (Zhang et al. 2015), β-hydroxyacyl-CoA dehydrogenase (β-HAD) (Alrob et al. 2014), and enoyl-CoA hydratase-1 (ECHS1) (Zhang et al. 2017) during β-oxidation. In addition, SIRT3 can activate acetyl-CoA carboxylase (ACC1) to promote fatty acid synthesis (Xu et al. 2020), which is a beneficial way of energy storage when nutrients are plentiful.

OXPHOS is the process by which electron transfer chain (ETC) converts the metabolites NADH and FADH2 to ATP (Nolfi-Donegan et al. 2020). There are five complexes involved in this process, which are labeled as complexes I to V. SIRT3 can deacetylate and activate the NADH dehydrogenase (ubiquinone) 1 alpha subcomplex 9 (NDUFA9) subunit on complex I (Ahn et al. 2008), succinate dehydrogenase flavoprotein (SDHA) subunit on complex II (Cimen et al. 2010), ubiquinol cytochrome c reductase core protein 1 (UQCRQ) subunit on complex III (Sun et al. 2019), cytochrome c oxidase-1 (COX-1) subunit on complex IV (Tu et al. 2019), and ATP synthase β subunit on complex V (Rahman et al. 2014), hereby promoting the generation of ATP. Furthermore, deacetylation of p53 by SIRT3 inhibits the expression-repressive effect of p53 on ND2 and ND4 genes, which encode key subunits of complex I (Lee et al. 2018). SIRT3 also activates leucine-containing protein 130 (LRP130), which promotes the process of OXPHOS (Liu et al. 2014). However, it has been reported that deacetylation of ceramide synthases 1 (CerS1), CerS2, and CerS6 by SIRT3 promotes ceramide accumulation and inhibits complex III activity (Novgorodov et al. 2016).

Amino acid metabolism can also generate small amounts of energy, and SIRT3 can deacetylate and activate glutamate dehydrogenase (GDH) (Choi et al. 2016). This enzyme facilitates the conversion of glutamate to alpha-ketoglutarate, which enters the TCA cycle for energy production. This process produces the toxic metabolite ammonia (NH_3_), and the urea cycle is the main metabolic route of intracellular NH_3_. Activation of ornithine transcarbamoylase (OTC) (Hallows et al. 2011) and carbamoyl phosphate synthase 1 (CPS1) (Li et al. 2016a, b) by SIRT3 in the urea cycle can promote urea synthesis and inhibit the toxic effects of NH_3_ on the body.

SIRT3 also has a partial effect on the metabolism of nonnutrients. SIRT3 can deacetylate and inhibit aldehyde dehydrogenase 2 (ALDH2) activity and thereby inhibit the conversion of acetaldehyde to acetate (Wei et al. 2013a, b), which may cause acetaldehyde toxicity. SIRT3 can also deacetylate acetyl-CoA synthase 2 (AceCS2) to convert acetate to acetyl-CoA (Hallows et al. 2006), and then participate in the TCA cycle.

### SIRT3 and mitochondrial dynamics

Mitochondrial dynamics include two groups of events: mitochondrial fusion and mitochondrial fission, and mitochondrial biogenesis and mitochondrial degradation, including mitophagy (Meyer et al. 2017). Together, these four processes maintain the stability of mitochondrial number, shape, and function in cells (Forte et al. 2021). SIRT3 regulates the activity of key enzymes in mitochondrial dynamics through deacetylation, thereby regulating the stabilization of mitochondrial function in cells.

Optic atrophy 1 (Opa1) and mitofusin (Mfn) are major proteins involved in mitochondrial fusion, namely in promoting fusion of the inner and outer mitochondrial membranes, respectively. There are two isoforms of Mfn, namely Mfn1 and Mfn2 (van der Bliek et al. 2013). SIRT3 can directly deacetylate Opa1 at lysine 926 and 931 and promote its activity (Samant et al. 2014). However, SIRT3 does not directly deacetylate Mfn, but it can deacetylate and activate liver kinase B1 (LKB1) (Pillai et al. 2010), thereby promoting mitochondrial fusion through the LKB1–AMP activated protein kinase (AMPK)–Mfn pathway (Dong et al. 2019; Fan et al. 2019; Wu et al. 2020a, b, c). The proteins involved in mitochondrial fission are mainly dynamin related protein 1 (DRP1) and mitochondrial fission protein 1 (Fis1). There is no direct deacetylation of these two proteins by SIRT3, and deacetylation of forkhead box O3a (FOXO3a) by SIRT3 can upregulate the expression levels of DRP1 and Fis1, which are involved in the regulation of mitochondrial dynamics (Tseng et al. 2013). However, one experiment has shown that reduced levels of SIRT3 in brain tissue do not affect DRP1 expression, but promote DRP1 phosphorylation and translocation from the cytoplasm to mitochondria, thereby promoting mitochondrial fission (Park et al. 2020). This is most likely mediated by the LKB1–AMPK pathway (Mao et al. 2022; Xue et al. 2019).

Peroxisome proliferator-activated receptor gamma coactivator-1alpha (PGC-1α) is a core protein that causes mitochondrial biogenesis, which can be positively regulated by the LKB1–AMPK–PGC-1α pathway, and participates in mitochondrial biogenesis through the PGC-1α– nuclear respiratory factor (NRF)–transcription factor A (TFAM) pathway; thus, deacetylation modification of LKB1 by SIRT3 also promotes mitochondrial biogenesis (Fu et al. 2012; Li et al. 2017). TFAM can activate RNA polymerase in mitochondria and directly promote transcription of the genome in mitochondria. In kidney tumor cells, SIRT3 deacetylates and activates TFAM, which in turn promotes mitochondrial biogenesis (Liu et al. 2018a, b, c). E3 ubiquitin ligase Parkin-mediated mitophagy is important for the maintenance of mitochondrial homeostasis and is an important clearance mechanism for mitochondria. This process is regulated by FOXO3a, and SIRT3 is one of the important regulatory enzymes of FOXO3a. FOXO3a is activated upon deacetylation and promotes Parkin expression (Ma et al. 2018; Yu et al. 2017).

### SIRT3 and oxidative stress

OS is an intracellular damage state, which is closely related to the occurrence and development of many diseases. Excessive intracellular production of reactive oxygen species (ROS) is an important cause of OS. The abnormal working state of the oxidative respiratory chain causes a large amount of electron leakage, which is one of the important reasons for the excess of ROS. The effects of SIRT3 on the five complexes in the oxidative respiratory chain can affect the production of ROS (Ahn et al. 2008; Cimen et al. 2010; Lee et al. 2018; Novgorodov et al. 2016; Sun et al. 2019; Tu et al. 2019). Impaired ROS scavenging is another cause of OS, and endogenous antioxidant enzymes can scavenge superoxide in cells to inhibit OS. SIRT3 directly regulates the activities of antioxidant enzymes such as manganese superoxide dismutase (MnSOD) (Qiu et al. 2010), catalase (CAT) (Wang et al. 2014), and peroxiredoxin3 (PRDX3) (Wang et al. 2020a, b) through deacetylation, thereby inhibiting OS. SIRT3 also indirectly activates MnSOD by mediating the deacetylation of FOXO3a and forkhead box O1 (FOXO1), promoting ROS scavenging (Tseng et al. 2014; Zhang et al. 2013). In addition, deacetylated FOXO3a also promotes CAT activity (Zhang et al. 2018a, b). ROS scavenging by glutathione peroxidase (Gpx) can also be enhanced by its deacetylation by SIRT3 (Yoon and Kim 2016). Glutathione (GSH) acts as a cofactor for Gpx to scavenge ROS, while nicotinamide adenine dinucleotide phosphate (NADPH) is a necessary factor to generate GSH. Deacetylation activation of IDH2 (Someya et al. 2010) and mitochondrial methylenetetrahydrofolate dehydrogenase/cyclohydrolase (MTHFD2) (Wan et al. 2020) by SIRT3 can promote NADPH production, thereby inhibiting OS. However, one study came to the opposite conclusion; i.e., that deacetylation of glutamate oxaloacetate transaminases2 (GOT2) by SIRT3 inhibits NADPH production, which in turn leads to tumor cell death under conditions of OS (Yang et al. 2015a, b).

### SIRT3 and apoptosis

The relationship between SIRT3 and apoptosis has only been explored in a few experiments. In general, inhibition of OS by SIRT3 can inhibit the occurrence of apoptosis. In addition, SIRT3 can regulate apoptosis by regulating the deacetylation of the following proteins. First, activation of glycogen synthase kinase 3β (GSK-3β) by SIRT3 can promote the expression of BCL2-associated protein X (Bax), which in turn promotes apoptosis (Song et al. 2016). he inhibitory effect of SIRT3 on apoptosis can be achieved by activating 8-oxoguanine DNA glycosylase 1 (OGG1) (Cheng et al. 2013), Ku70 (Sundaresan et al. 2008), and 17-β-hydroxysteroid dehydrogenase 10 (HSD17B10) (Liu et al. 2020a, b, c), and inhibiting the activity of CypD (Liu et al. 2018a, b, c). OGG1 can repair DNA damage caused by OS; Ku70 can inhibit Bax-induced apoptosis; both the increased activity of HSD17B10 and the decreased activity of CypD inhibit mitochondrial dysfunction.

### SIRT3 and autophagy

Autophagy is a highly conserved eukaryotic cell cycle process. Subcellular structures such as organelles are degraded under the action of certain mechanisms to form decomposition products that can be recycled. Autophagy thus plays an important role in maintaining cellular homeostasis (Parzych and Klionsky 2014). In addition to regulating mitophagy by regulating the activity of FOXO3a, SIRT3 can also regulate autophagy through the LKB1–AMPK–mammalian/mechanistic target of the rapamycin (mTOR) axis. mTOR inhibits autophagy in general, and activated AMPK can inhibit mTOR activity, so deacetylation of LKB1 by SIRT3 can promote autophagy (Zhang et al. 2018a, b). The Atg12–Atg5–Atg16 complex is an important regulatory system during autophagy (Yang and Klionsky 2009), and deacetylation of ATG5 by SIRT3 ensures autophagosome maturation (Liu et al. 2018a, b, c).

## Chronic neurodegenerative diseases

**Fig. 1:**
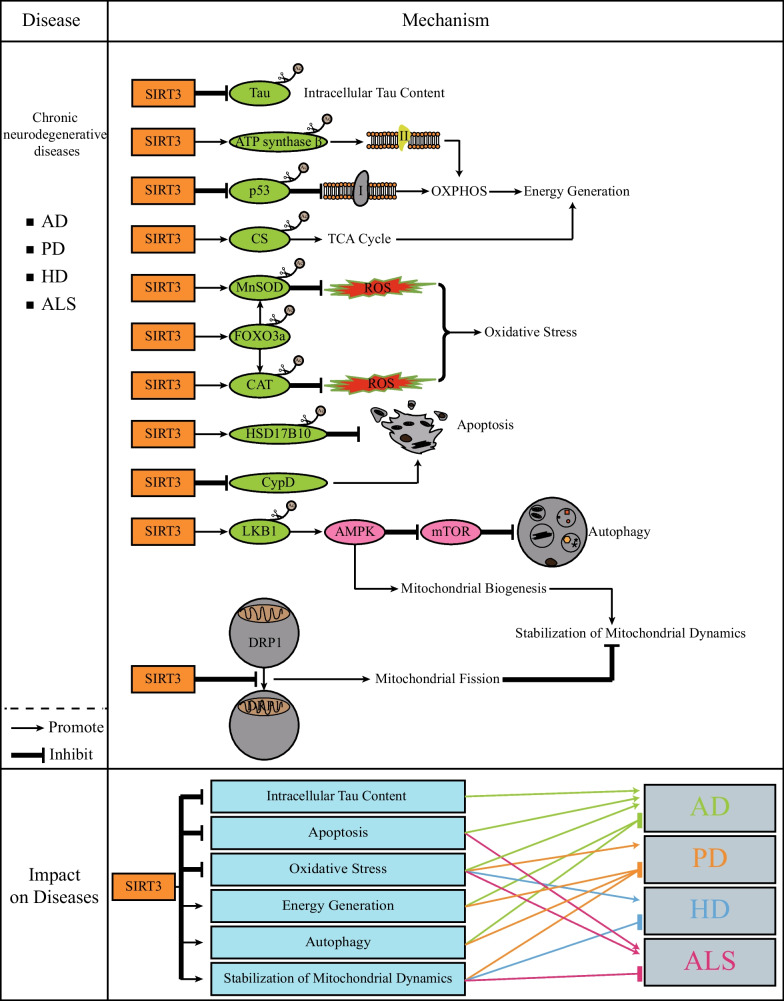
*SIRT3 and chronic neurodegenerative diseases.* The occurrence and development of chronic neurodegenerative diseases are related to the accumulation of intracellular toxic substances, programmed cell death and mitochondrial homeostasis. SIRT3 activates a range of substrates by deacetylation to reduce intracellular Tau content, promote energy production, inhibit OS and apoptosis, and promote autophagy. In addition, SIRT3 also maintains stable mitochondrial dynamics. The green proteins represent the substrates of SIRT3. Brown circles represent acetyl group

Chronic neurological diseases are closely related to age and mostly occur in the elderly. In this section, we mainly focus on Alzheimer's disease (AD), Parkinson's disease (PD), Huntington's disease (HD) and amyotrophic lateral sclerosis (ALS) (Fig. 1).

### SIRT3 and Alzheimer's disease

AD is a very common chronic neurodegenerative disease characterized by memory impairment, executive dysfunction, and personality and behavior abnormalities, which seriously affect the quality of life of the elderly in the late stage. The etiology of AD is very complex and has not yet been fully elucidated. OS, neuronal apoptosis, inflammation, and cellular senescence can all promote the occurrence and development of AD.

It has been found that the level of SIRT3 in mitochondria of the cerebral cortex of AD mice is decreased (Yang et al. 2015a, b) and that the level of SIRT3 mRNA in the brain tissue of AD patients is reduced (Song et al. 2020). These results indicate that the presence of SIRT3 may be very important to inhibit the occurrence and development of AD. In the early stage of AD, there is an excessive accumulation of Tau protein in the brain tissue (Li et al. 2019). However, SIRT3 can deacetylate Tau and reduce its protein content (Li et al. 2019; Yin et al. 2018), which may help inhibit the further development of AD (Villemagne et al. 2015). SIRT3 can also inhibit the acetylation level of p53, thereby restoring the normal expression levels of ND2 and ND4 genes in the brain tissue. This is vital for maintaining the normal progress of mitochondrial OXPHOS and inhibiting AD caused by neuronal damage (Lee et al. 2018). Reducing the content of ROS in the cell may be an effective method to treat AD. One of the most important substrates of SIRT3 is MnSOD, and the activity of MnSOD is enhanced after deacetylation. MnSOD can convert excessive ROS into hydrogen peroxide (H_2_O_2_). H_2_O_2_ is converted into harmless oxygen and water under the action of CAT. This function of SIRT3 has been confirmed in multiple experiments (Li et al. 2020; Liu et al. 2020a, b, c; Ramesh et al. 2018). SIRT3 can promote the deacetylation and activity of 17-β-hydroxysteroid dehydrogenase 10 (HSD17B10), thereby promoting the antiapoptotic ability of cells (Liu et al. 2020a, b, c). Although there is currently no evidence that the effect of SIRT3 on HSD17B10 can inhibit AD, the inhibition of HSD17B10 activity by amyloid-β (Aβ) can lead to neuronal dysfunction associated with AD (Oppermann et al. 1999; Yan et al. 1997). Therefore, it is credible that HSD17B10 mediates the inhibitory effect of SIRT3 on AD. SIRT3 can also inhibit neuronal apoptosis by inhibiting the expression of CypD, and thus alleviates the symptoms of AD (Jiang et al. 2017). In addition, the inhibitory effect of SIRT3 on AD can be achieved by promoting the canonical autophagy pathway LKB1–AMPK–mTOR (Shu et al. 2020; Zhang et al. 2020a, b, c, d).

### SIRT3 and Parkinson’s disease

Among neurodegenerative diseases, PD is the second by incidence (Risiglione et al. 2021; Tysnes and Storstein 2017). PD is characterized by progressive degeneration of dopaminergic neurons in the substantia nigra striatum (Liu et al. 2015). The main clinical manifestations of the disease are motor dysfunction including tremors at rest, rigidity, postural instability, and bradykinesia (Huang et al. 2021; Li et al. 2021a, b; Tolosa et al. 2006).

In several chemical drug trials, it has been found that SIRT3 agonists such as theacrine (Duan et al. 2020) and trans-(-)-ε-viniferin (ε-viniferin) (Zhang et al. 2020a, b, c, d) can inhibit the occurrence and development of PD. When SIRT3 inhibitors such as dipeptidyl peptidase-4 (DPP-4), P2X7 purinoceptors (P2X7R) (Jamali-Raeufy et al. 2020), and miR-494-3p (Geng et al. 2018) are inhibited, PD and related clinical symptoms improve. Hence, SIRT3 has a positive effect on PD inhibition.

In current research, the inhibitory effect of SIRT3 on PD is mainly achieved by inhibiting the accumulation of ROS in cells and consequent inhibition of OS (Dai et al. 2014), which is similar to the effect of SIRT3 on AD. SIRT3 can promote the activity of MnSOD (Jiang et al. 2019; Zhang et al. 2016) as well as the expression level of MnSOD in cells by promoting the deacetylation level and activity of FOXO3a (Rangarajan et al. 2015). FOXO3a can also promote the expression of CAT (Rangarajan et al. 2015). Therefore, under this series of actions, SIRT3 strongly eliminates the content of ROS in neuronal cells. In addition, it has been shown that the promotion of autophagy by SIRT3 can inhibit the occurrence and development of PD (Zhang et al. 2018a, b). SIRT3 can increase the deacetylation level and activity of LKB1 (Woods et al. 2003), which in turn promotes the phosphorylation level and activity of AMP activated protein kinase (AMPK) (Pillai et al. 2010) and inhibits the activity of mTOR, thereby promoting autophagy (Jung et al. 2010). SIRT3 also promotes the activities of ATP synthase β (Zhang et al. 2016) and CS (Cui et al. 2017) to promote OXPHOS and the TCA cycle, respectively. The resulting large amount of energy inhibits neuronal damage caused by insufficient energy. Recent studies have shown that SIRT3's inhibition of DRP1 phosphorylation can maintain normal mitochondrial dynamics, which can positively regulate the treatment and prognosis of PD (Park et al. 2020).

### SIRT3 and Huntington's disease

Unlike AD and PD, the incidence of HD is low and the pathogenesis is clearer. The disease is an autosomal dominant genetic disease (Wild and Tabrizi 2017). The mutated chromosomes can cause the neurotoxic mutant huntingtin (mHTT) to accumulate in the body (Ross and Tabrizi 2011). The most common clinical sign of the disease is dyskinesia, and patients often show weird dance-like movements (Jimenez-Sanchez et al. 2017). In addition, many patients have different levels of psychiatric symptoms, such as depression and obsessive–compulsive disorder (Rosenblatt 2007). This may be caused by the strange behavior, or it may be caused by the organic disease directly caused by mHTT.

At present, treatments for HD related to SIRT3 mostly focus on how to maintain the normal mitochondrial biogenesis because abnormal mitochondrial biogenesis is one of the important factors in the development of HD. After SIRT3 is activated, the LKB1–AMPK pathway is also activated and enhances mitochondrial biogenesis (Fu et al. 2012). Moreover, AMPK can also promote the activity of SIRT3 by increasing the NAD^+^/NADH ratio, thereby forming a positive feedback pathway (Duan et al. 2016). In addition, the inhibitory effect of SIRT3 on ROS can inhibit the damage of mHTT to neurons (Fu et al. 2012). It has also been shown that the ability of SIRT3 to inhibit the accumulation of DRP1 in mitochondria and thus inhibit mitochondrial fission contributes to mitochondrial elongation, which in turn promotes cell viability (Naia et al. 2021; Oliver and Reddy 2019). Although there are few related studies, the agonist of SIRT3 used in the above two studies is viniferin, and viniferin has a good inhibitory effect on HD.

### SIRT3 and amyotrophic lateral sclerosis

ALS is a neurodegenerative disease for which the cause has not yet been identified. The disease is characterized by the degeneration of motor neurons (MN) in the brain and spinal cord, and the resulting muscle dysfunction such as limb weakness and difficulty swallowing (Taylor et al. 2016). Most patients eventually die from respiratory failure caused by respiratory muscle dysfunction (Brown and Al-Chalabi 2017). Although the incidence of the disease is relatively low, patients endure great pain during the illness. Therefore, it is necessary to improve the clinical symptoms and cure the disease.

At present, the relationship between SIRT3 and ALS has been relatively poorly studied. However, since mitochondrial morphology is altered in ALS disease models (Magrané et al. 2009; Sasaki and Iwata 2007), inhibition of mitochondrial fission by SIRT3 may inhibit the development of ALS (Song et al. 2013). CypD inhibition by SIRT3 prevents the transition of mitochondrial permeability and thus inhibits apoptosis, which also plays an important role in the inhibition of ALS (Song et al. 2013). MN of ALS patients has specific metabolic characteristics such as reduced mitochondrial respiration and elevated glycolysis. Therefore, the reversal of the clinical phenotype of ALS by SIRT3 agonists is inseparable from the function of SIRT3 to promote OXPHOS (Hor et al. 2021). This is one of the reasons why SIRT3 agonists are the preferred treatment strategy for ALS in some treatment regimens (Harlan et al. 2020).

## Acute neurodegenerative diseases

**Fig. 2:**
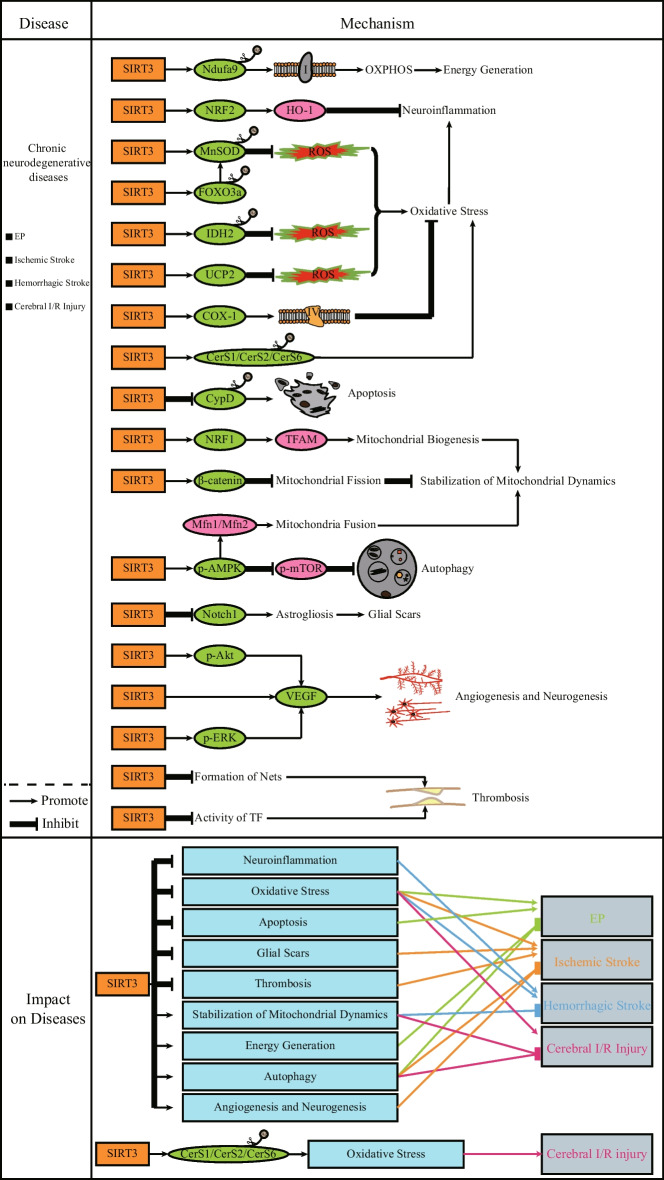
*SIRT3 and acute neurodegenerative diseases.* In the vast majority of cases, SIRT3 has a positive effect on the treatment of acute neurodegenerative diseases. Inhibition of apoptosis and OS, as well as promotion of energy production, autophagy, and stabilization of mitochondrial dynamics by SIRT3 can also inhibit the progression of acute neurodegenerative diseases. SIRT3 can also alleviate the clinical manifestations of the diseases by inhibiting neuroinflammation, glial Scars and thrombosis, promoting angiogenesis and nneurogenesis. However, it is worth noting that deacetylation of CerS1, CerS2, and CerS6 by SIRT3 accelerates the accumulation of ceramides, which induces OS that further exacerbates cerebral I/R injury. The green proteins represent the substrates of SIRT3. Brown circles represent acetyl group

Acute neurological diseases are mostly related to abnormal discharge of cerebral nerves and cerebral vascular lesions, and are often accompanied by serious sequelae. In this section, we will mainly discuss how SIRT3 regulates its substrates to regulate the occurrence and development of acute neurological diseases (Fig. 2).

### SIRT3 and epilepsy (EP)

Simply put, EP is a disease caused by excessive discharge of central nervous system neurons (Marchi et al. 2014). The etiology of the disease is very complex, and EP is generally divided into the following four categories: idiopathic, symptomatic, provoked, and cryptogenic (Shorvon 2011). Effective treatment measures depend on the cause of EP.

The expression of SIRT3 is reduced in a chronic EP disease model (Gano et al. 2018), which suggests that SIRT3 may become a target protein for the treatment of EP. SIRT3 can promote the activity of FOXO3a, MnSOD, and IDH2, and can also increase the expression of uncoupling protein 2 (UCP2) in neurons, which all contribute to the inhibition of neuronal OS damage (Cho et al. 2019; Gano et al. 2018; Hasan-Olive et al. 2019). CypD is deacetylated by SIRT3 and its activity is inhibited, which limits the opening of mitochondrial membrane permeability transition pore (mPTP) and inhibits neuronal apoptosis, thereby protecting neurons from excitotoxicity (Cheng et al. 2016). The promotion of autophagy by SIRT3 during seizures also reduces brain damage, but it has not been determined whether the relevant mechanism involves the LKB1–AMPK–mTOR pathway, which is closely linked to autophagy (Wang et al. 2018; Wu et al. 2020a, b, c). The deacetylation of NDUFA9 by SIRT3 can promote the activity of complex I, thereby inhibiting mitochondrial dysfunction and promoting ATP production (Ahn et al. 2008; Gano et al. 2018). OS damage and abnormal energy metabolism are important mechanisms leading to the occurrence and deterioration of EP (Pauletti et al. 2019; Pearson-Smith and Patel 2017). Therefore, SIRT3 agonists may become effective drugs for the treatment of EP. In addition, inflammation seriously affects the occurrence and development of EP (Vezzani et al. 2011), and SIRT3 can also effectively inhibit the inflammatory response in a variety of ways (Almalki et al. 2021; Dikalova et al. 2020; Huang et al. 2019; Palomer et al. 2020; Song et al. 2019). However, the relationship between the three and related mechanisms has not yet been fully clarified.

### SIRT3 and ischemic stroke

When the cerebral blood vessels are ruptured or blocked, blood cannot flow into the brain in time, so the brain tissue is damaged to different degrees, which is known as a stroke. Depending on the cause, stroke is divided into ischemic stroke and hemorrhagic stroke (Broderick et al. 2017). The incidence of the former is greater than that of the latter. Regardless of the type of stroke, if patients are not treated in time, they often suffer from serious sequelae. At present, due to the rapid progression of the disease, the prevention of stroke occupies a more important position than the treatment of stroke. However, when patients are not treated in time, powerful and effective drugs can alleviate the brain damage caused by stroke, which provides great help to improve the patients' quality of life.

Several investigators have reported that SIRT3 expression in mouse hippocampal neurons is decreased after acute ischemic stroke and that acute ischemia-induced neuronal damage is attenuated under conditions of SIRT3 overexpression (Fan et al. 2021). This suggests that SIRT3 may contribute to the treatment of nerve damage caused by an ischemic stroke. As previously mentioned, SIRT3 promotes the phosphorylation level of AMPK and then inhibits the phosphorylation level of mTOR to promote autophagy. This mechanism also plays a protective role against ischemia-induced brain injury (Dai et al. 2017; Li et al. 2021a, b). However, it remains unknown whether this effect involves the activation of LKB1 by SIRT3 in this disease condition. Activated AMPK can also activate SIRT3 by activating PGC-1α, which in turn forms a positive feedback pathway (Gao et al. 2018). The function of SIRT3 to activate FOXO3a and MnSOD to inhibit OS can also alleviate the damage caused by cerebral ischemia (Wang et al. 2020a, b; Wang et al. 2015; Yin et al. 2015). Glial scars produced after a cerebral ischemic injury can inhibit neuronal repair (Bao et al. 2012; Barres 2008; Jeong et al. 2012; Ridet et al. 1997). Inhibition of Notch1 expression by SIRT3 inhibits astrocyte activation, which in turn inhibits glial scar formation (Yang et al. 2017). However, excessive inhibition of the Notch signaling pathway may inhibit SIRT3 (Guo et al. 2020). Therefore, it is very important to adjust the balance between SIRT3 and Notch1. One of the sequelae of cerebral ischemic injury is cognitive dysfunction (CD). SIRT3 can also promote the phosphorylation level of advanced protein kinase B (AKT) and extracellular signal-regulated kinases (ERK) and the expression of vascular endothelial growth factor (VEGF), thereby promoting neurogenesis and angiogenesis in the injured area. In addition, phosphorylated AKT and ERK can also promote the expression of VEGF (Yang et al. 2018a, b). SIRT3 can also promote the transfer of microglia to cerebral ischemic areas by promoting the expression of fractalkine receptor (CX3CR1) (Cao et al. 2019). However, due to different disease conditions, microglia can both relieve ischemic damage and promote ischemic damage (Block et al. 2007; Fu et al. 2014; Nakajima and Kohsaka 2004). Therefore, the role of SIRT3 in this aspect needs to be further explored.

From the perspective of prevention, SIRT3 has an inhibitory effect on neutrophil extracellular traps (NETs) and plasma tissue factor (TF). This can inhibit the formation of thrombi, thereby reducing the incidence of ischemic brain injury (Gaul et al. 2018).

### SIRT3 and hemorrhagic stroke

Intracerebral hemorrhage (ICH) and subarachnoid hemorrhage (SAH) are the two most representative subtypes of hemorrhagic stroke (Luo et al. 2019; Zhao et al. 2018a, b). The former is characterized by the accumulation of blood in the brain tissue (Zhao et al. 2018a, b), which accounts for 10%–20% of all stroke events worldwide (Qureshi et al. 2009; Sporns et al. 2021; van Asch et al. 2010); the latter is characterized by the accumulation of blood in the subarachnoid space (Zhao et al. 2018a, b) and accounts for 5%–15% of all strokes (Liao et al. 2020).

Inhibition of ROS production by SIRT3 inhibits both OS injury and the expression of NOD-like receptor family pyrin domain containing 3 (NLRP3) inflammasome and interleukin-1beta (IL-1β) and the resulting neuroinflammation (Ma et al. 2014; Zheng et al. 2018; Zhou et al. 2010). Inhibition of neuroinflammation by SIRT3 can also be achieved by activating NRF2. NRF2 promotes the expression of heme oxygenase-1 (HO-1), which in turn inhibits the production of inflammatory factors such as tumor necrosis factor-α (TNF-α) and IL-1β (Dai et al. 2022). Furthermore, activation of the NRF1–TFAM pathway by SIRT3 promotes mitochondrial biogenesis, which maintains stable mitochondrial dynamics (Zheng et al. 2018). The above effects can effectively improve secondary brain injury (SBI) caused by ICH (Zheng et al. 2018).

Similar to the impact of SIRT3 on ICH, SIRT3 also has a protective effect on SAH-induced brain damage (Huang et al. 2016). Some researchers have found that SIRT3 in SAH model mice is inhibited, and the ability of neurons to resist OS is reduced (Zhang et al. 2020a, b, c, d). After SIRT3 is activated, it can be found that the degree of neuronal OS damage and the level of neuronal apoptosis are reduced (Wu et al. 2020a, b, c; Yang et al. 2018a, b; Zhang et al. 2019). SIRT3 can also promote the expression of Mfn1 and Mfn2 by activating AMPK, thereby promoting mitochondrial fusion and maintaining the normal mitochondrial morphology (Wu et al. 2020a, b, c). This plays an important role in maintaining the normal function of mitochondria.

### SIRT3 and cerebral ischemia–reperfusion injury

As mentioned above, cerebral ischemia can cause serious damage, and the degree of damage is closely related to the time of ischemia (Carden and Granger 2000; Kloner et al. 1974; Raedschelders et al. 2012). Therefore, timely blood perfusion to the ischemic site can ensure the survival of the ischemic tissue (Wu et al. 2021). However, the damage to ischemic tissues undergoing reperfusion therapy in some experiments was abnormally enhanced (Hearse et al. 1973; Reimer et al. 1977). This is a kind of OS damage caused by the entry of active oxygen into the dredged parts (Granger and Kvietys 2015). Therefore, the elimination of active oxygen has become an effective means of treating this kind of disease.

Increasing the content and activity of SIRT3 can inhibit cerebral I/R injury (Gao et al. 2020; Liu et al. 2020a, b, c; Su et al. 2017). The activation of MnSOD by SIRT3 can also alleviate the OS damage of the brain tissue caused by I/R (Liu et al. 2021a, b). SIRT3 can inhibit the phosphorylation level and activity of β-catenin, thereby inhibiting excessive mitochondrial fission and maintaining the stability of mitochondrial dynamics (Zhao et al. 2018a, b). SIRT3 can also promote the deacetylation level of COX-1, which can improve OS after I/R (Tu et al. 2019). In addition, the promotion of autophagy by SIRT3 mediated by the AMPK–mTOR pathway can also inhibit I/R injury, but this function is achieved by removing damaged nerve cells rather than by inhibiting OS (Chen et al. 2021a, b). However, an experiment conducted in 2016 showed that SIRT3 is activated by a currently unknown mechanism and increases the deacetylation level and activity of CerS1, CerS2, and CerS6 after cerebral ischemia–reperfusion. This leads to the accumulation of ceramides. High levels of ceramides can lead to OS damage (Novgorodov et al. 2016). This is completely contrary to the previous conclusion, which suggests that the protective effect of SIRT3 on the nervous system may depend on a state of balance. Once this balance is broken, SIRT3 exerts a negative effect. The specific reasons are expected to be verified in future experiments.

## SIRT3 and CD caused by non-neurodegenerative diseases

Almost all neurodegenerative diseases can cause CD, especially PD and AD. However, some nonneurological diseases and clinical operations can also cause CD. There are some differences between the two. Therefore, it is necessary to explain this part separately.

**Fig. 3:**
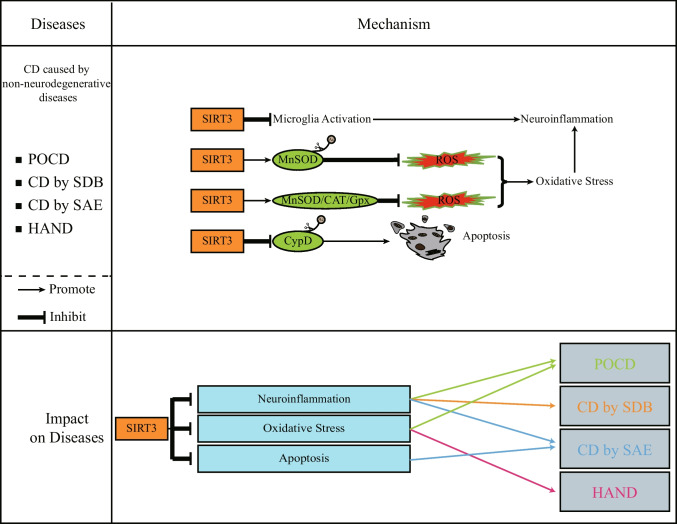
*SIRT3 and CD caused by non-neurodegenerative diseases.* SIRT3 can alleviate CD caused by non-neurodegenerative diseases. This is achieved through inhibition of neuroinflammation, OS and apoptosis. SIRT3 can inhibit the activation of microglia and thus inhibit neuroinflammation. However, the mechanism of this activation has not yet been elucidated. The green proteins represent the substrates of SIRT3. Brown circles represent acetyl group

Postoperative delirium and cognitive dysfunction (POCD) is a common complication that occurs in elderly patients after surgery (Deiner and Silverstein 2009), which can lead to an increase in postoperative mortality. This phenomenon is related to the OS response of mitochondria (Netto et al. 2018). SIRT3 promotes the inhibition of OS by activating MnSOD, and the consequent inhibition of neuroinflammation can alleviate POCD (Bajwa et al. 2019; Liu et al. 2021a, b; Ye et al. 2019). In addition, the inhibitory effect of SIRT3 on neuroinflammation plays an important role in alleviating sleep-disordered breathing (SDB)-induced CD (Lin et al. 2020). Through acetylome analysis, SIRT3's deacetylation of brain mitochondrial proteins can also alleviate CD induced by metabolic syndrome (MetS) (Tyagi et al. 2018). Infection can also cause CD. Deacetylation of CypD by SIRT3 attenuates sepsis-associated encephalopathy (SAE)–induced CD through the inhibition of apoptosis and neuroinflammation (Sun et al. 2017). SIRT3 can inhibit the decrease in the expression of antioxidant enzymes MnSOD, CAT, and Gpx caused by human immunodeficiency virus (HIV) transactivator of transcription (TAT), thereby inhibiting OS-induced microglia senescence, and improving HIV-associated neurocognitive disorders (HAND) (Thangaraj et al. 2021). Thus, SIRT3 improves CD caused by different reasons (Fig. 3). The modulation of SIRT3 will help to improve the quality of life of related patients.

## SIRT3 and rehabilitation training

Neurological diseases are often incurable and irreversible. Therefore, it is critical to alleviate the progression of related diseases and complications through some treatment. This can greatly improve the patient's quality of life. Several types of rehabilitation training mediated by SIRT3 are briefly introduced here.

### Effects of physical activity on SIRT3 and related diseases

A sedentary lifestyle is an important risk factor for various diseases, such as cardiovascular disease, diabetes, and neurological diseases (Korta et al. 2019). Reasonable exercise can improve the prognosis of most diseases and promote human health (Koltai et al. 2018; Vargas-Ortiz et al. 2015). A comparative study conducted in 2015 compared the cognitive function of athletes who exercised regularly with that of nonathletes who were sedentary and found that athletes had better memory and reaction times (Zhao et al. 2016). The results of another similar study were also reported in 2019. The results of the experiment showed that the average cognitive ability of rugby players is stronger than that of low-intensity groups of the same age. After comparing the blood samples of the above two groups of people, the expression levels of SIRT3, CAT, and SOD1 in the low-activity group were shown to be significantly lower than those in the rugby players. This suggests that the beneficial effects of exercise on neurological diseases may be related to the anti-OS effect of SIRT3 (Corpas et al. 2019).

Some researchers have used a treadmill to perform exercise training experiments on AD model mice. The results showed that compared with the non-exercise group, the symptoms of the mice after 20 weeks of standard training were relieved; additionally, the content of SIRT3 was increased and the acetylation level of MnSOD and OGG1 were decreased in the hippocampus. SIRT3 activates MnSOD and OGG1 to inhibit OS and repair DNA damage, which is an important mechanism by which physical exercise alleviates AD symptoms (Bo et al. 2014).

SIRT3 may also mediate the remission of physical exercise in neurological disorders caused by severe metabolic diseases. After performing aerobic interval training (AIT) on mice, we found that appropriate activity can effectively improve CD caused by obesity (Shi et al. 2018). This is not only related to the inhibition of OS by SIRT3 but also the inhibition of obesity by SIRT3. Aerobic training (AT) promotes the expression of SIRT3, which activates PGC-1α and key enzymes in the metabolism of substances. Therefore, the process of fatty acid oxidation and OXPHOS is accelerated, and the fat mass can be effectively reduced (Karvinen et al. 2016; Vargas-Ortiz et al. 2018).

### Effects of caloric restriction (CR) on SIRT3 and related diseases

CR is a nutritional intervention that reduces energy intake by 25%–30% while maintaining normal energy requirements (Pignatti et al. 2020). Numerous studies have demonstrated that CR can suppress the clinical manifestations of aging-related diseases and prolong lifespan (Barger et al. 2015; Kobayashi et al. 2017; Qiu et al. 2010; Someya et al. 2010; Wegman et al. 2015; Yu et al. 2018). During CR, the low energy intake favors a catabolic state; a large amount of acetyl-CoA is produced from the fatty acid metabolism process; mitochondrial proteins are in a hyperacetylation state. Therefore, to balance the levels of acetylation and deacetylation in cells, the activities and expression levels of various deacetylases, including SIRT3, are increased (Silaghi et al. 2021).

Although this is a compensatory change, the body does not suffer the damage caused by energy deficiency because the regulation of downstream proteins by the compensatory activation of deacetylases can fully compensate for this part of energy loss (Liu et al. 2014; North and Sinclair 2007; Shimazu et al. 2010). Conversely, the compensatory activation of deacetylases additionally gives the body a variety of protective measures. Intermittent fasting (IF)–induced elevation of SIRT3 protects neurons from excitotoxic damage in animal models of EP and AD. The increased activity of MnSOD after being deacetylated by SIRT3 inhibits the accumulation of ROS in cells. In this case, GABAergic tone is enhanced, thereby protecting neurons (Liu et al. 2019). The inhibition of neuroinflammation by IF also effectively protects the normal function of the brain tissue after ICH (Dai et al. 2022). In addition, studies on SIRT3 knockout mice have shown that they are more susceptible to excitotoxicity. At this time, mitochondria in brain tissue are more prone to mitochondrial permeability transition (MPT), and CypD is in a hyperacetylated state (Cheng et al. 2016). Therefore, deacetylation of CypD by the compensatory activation of SIRT3 under CR conditions inhibits mitochondrial permeability, thereby promoting mitochondrial calcium retention, which in turn inhibits neurological diseases caused by excitotoxic substances and calcium overload (Amigo et al. 2017). In addition to this, a high-fat–based and low-carbohydrate–based ketogenic diet (KD) contributes to the treatment and prognosis of refractory EP through compensatory mechanisms (Hasan-Olive et al. 2019).

In addition, although the subjects in some studies did not have neurological diseases, exercise and CR were shown to inhibit OS (Andrianova et al. 2020; Donniacuo et al. 2019; Jang et al. 2012; Jiang et al. 2014; Liang et al. 2013; Shi et al. 2005; Tsukiyama et al. 2017), inhibit inflammation (Traba et al. 2017, 2015), and promote autophagy (Li et al. 2018) through SIRT3. This is expected to provide theoretical support for future research on the relationship between rehabilitation training, SIRT3, and neurological diseases. Moreover, the aforementioned exercise methods and diet management are more or less different in different studies. Therefore, determining an appropriate range and selecting appropriate rehabilitation methods for different neurological diseases may be the focus of future research.

## Conclusion

To date, the morbidity and mortality of various neurological diseases are high, which is a global public health problem. However, because the pathogenesis of neurological diseases has not been fully clarified, various treatment measures have not had good therapeutic effects. Hence, a comprehensive understanding of neurological disease pathogeneses is necessary. In recent years, numerous experiments have shown that post-translational modifications are inseparable from the pathogenesis of neurological diseases. As a deacetylase, SIRT3 actively regulates the occurrence and development of neurological diseases through the regulation of downstream proteins. SIRT3 mainly inhibits various types of neurological diseases. This is achieved through the regulation of physiological activities such as OS, apoptosis, mitochondrial dynamics, and material metabolism by SIRT3. However, only one report has pointed to the deleterious effects of SIRT3 in nonneoplastic neurological diseases (Novgorodov et al. 2016). The accumulation of ceramides induced by SIRT3 is an important cause of cerebral ischemia–reperfusion injury, which is achieved through the activation of deacetylation of CerS1, CerS2, and CerS6 by SIRT3. In addition, the negative effects of SIRT3 on neurological diseases are mainly manifested in tumors because SIRT3 is ultimately responsible for the survival of nervous system cells through various forms of regulation. However, this kind of regulation acts on tumor cells to promote the proliferation and migration of cancer. For example, SIRT3 can promote the survival and invasion of glioma by inhibiting OS. Thus, for patients who have multiple diseases at the same time, establishing a clinical treatment method that correctly controls the dosage of SIRT3 activators and inhibitors to ensure an appropriate effect of SIRT3 remains challenging.

## Data Availability

All data generated or analysed during this study are included in this published article.
